# Supplementary material for: Patient-derived tumor organoids for personalized medicine in a patient with rare hepatocellular carcinoma with neuroendocrine differentiation: a case report
Source: Commun Med (Lond). 2022 Jul 1;2:80. doi: 10.1038/s43856-022-00150-3 (PMC9249908; doi:10.1038/s43856-022-00150-3)
Supplement: Supplementary file 1 — Description of Additional Supplementary Files [file 43856_2022_150_MOESM1_ESM.pdf]

Description of Additional Supplementary Files

File Name: Supplementary Data 1

Description: List of somatic mutations identified in the tumor and the matched organoids by whole-exome sequencing.

File Name: Supplementary Data 2

Description: Patient characteristics of all organoid lines.

File Name: Supplementary Data 3

Description: Source data IC50 values for all individual organoid lines.

File Name: Supplementary Data 4

Description: Source data of drug responses
