# Supplementary material for: Patient-derived tumor organoids for personalized medicine in a patient with rare hepatocellular carcinoma with neuroendocrine differentiation: a case report
Source: Commun Med (Lond). 2022 Jul 1;2:80. doi: 10.1038/s43856-022-00150-3 (PMC9249908; doi:10.1038/s43856-022-00150-3)
Supplement: Supplementary file 2 — Supplementary Information [file 43856_2022_150_MOESM2_ESM.pdf]

SUPPLEMENTARY INFORMATION

Patient-derived tumor organoids for personalized medicine in a rare case of hepatocellular carcinoma with neuroendocrine differentiation.

Marie-Anne Meier, Sandro Nuciforo, Mairene Coto-Llerena, John Gallon, Matthias S. Matter, Caner Ercan, Jürg Vosbeck, Luigi M. Terracciano, Savas D. Soysal, Daniel Boll, Otto Kollmar, Raphaël Delaloye, Salvatore Piscuoglio, and Markus H. Heim.

Table of contents

Supplementary Figure 1 2
Supplementary Figure 2 4
Supplementary Figure 3 6
Supplementary Figure 4 8
Supplementary References 10

Supplementary Figure 1

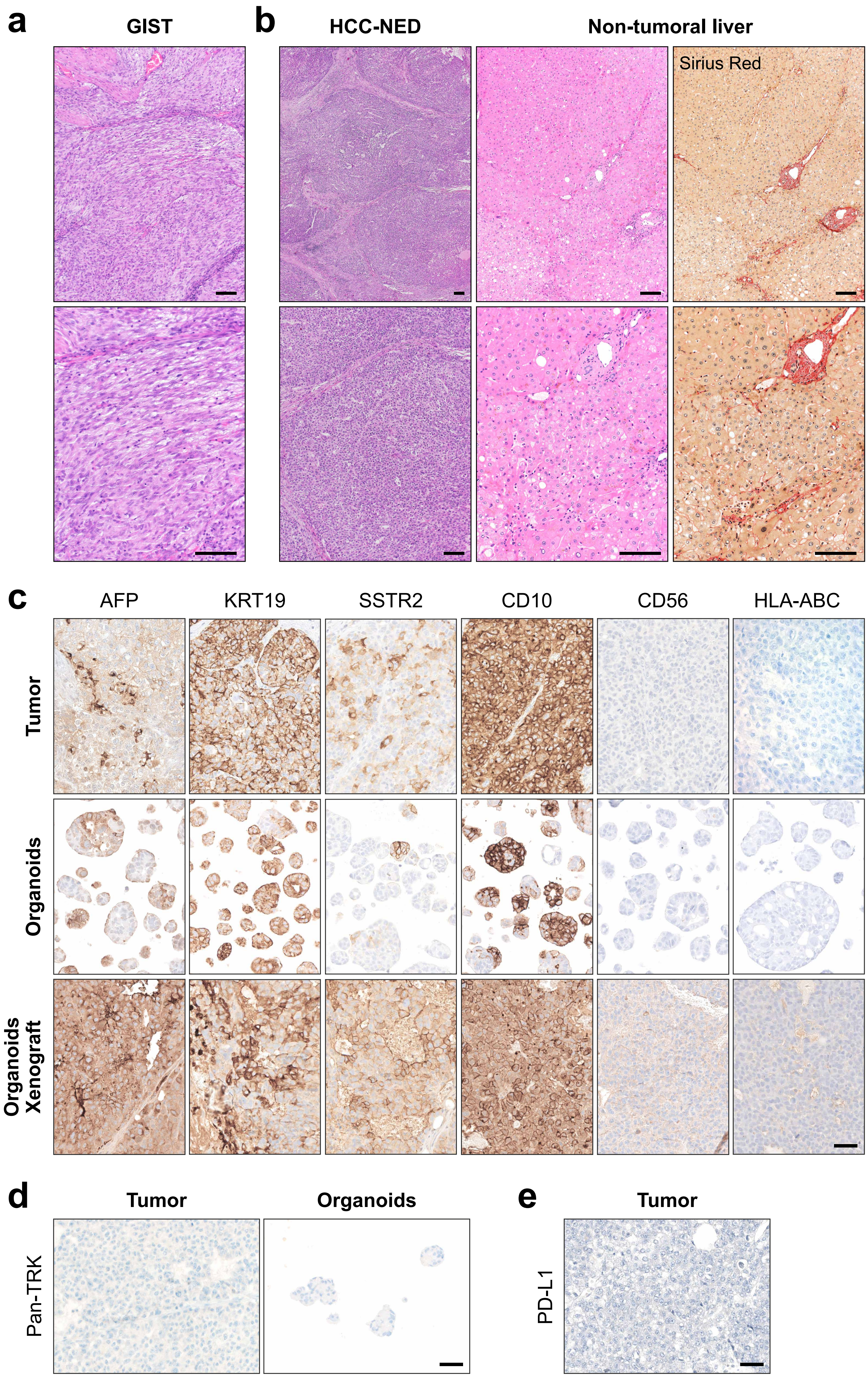

Supplementary Figure 1. **Histological features of the GIST, the non-tumoral liver as well as the primary tumor with its corresponding organoids and organoid-derived xenograft. a)** Representative images of the histopathological findings in the H&E (hematoxylin and eosin) stain of the GIST. Scale bar: 100 µm. GIST, gastrointestinal stromal tumor. **b)** Representative images of the histological findings in the H&E stain of the HCC as well as the H&E and Sirius Red stains of the adjacent non-tumoral liver. Scale bar: 100 µm. HCC-NED, hepatocellular carcinoma with neuroendocrine differentiation. **c)** Representative immunohistochemistry images of tumoral and hepatobiliary markers and their expression in corresponding organoid and xenograft tissues. The primary tumor as well as its organoids and the xenograft lack the expression of the Human Leucocyte Antigen (HLA)-ABC. Scale bar: 100 µm. AFP, Alpha Fetoprotein; CD10, Cluster of Differentiation 10; CD56, Cluster of Differentiation 56; KRT19, Keratin 19; SSTR2, Somatostatin Receptor 2. **d)** Representative images of Pan-TRK (Tropomyosin Receptor Kinase) immunohistochemistry on tumor tissue and its organoids. Scale bar: 100 µm. **e)** Representative images of PD-L1 (Programmed Death-Ligand 1) immunohistochemistry on tumor tissue. Scale bar: 100 µm.

Supplementary Figure 2

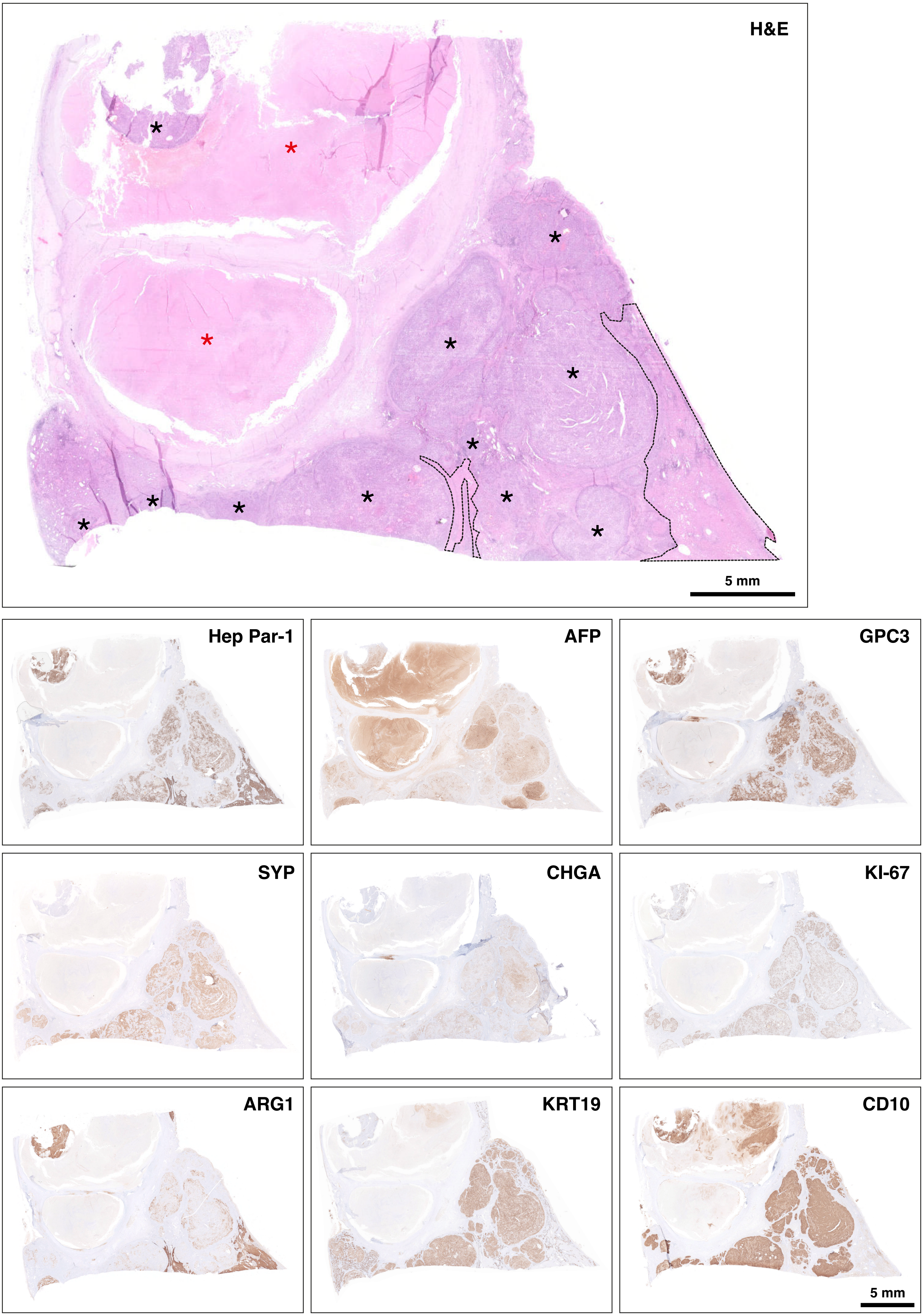

Supplementary Figure 2. Immunohistochemical characterization of HCC-NED tumor. Low magnification images of the tumor tissue. H&E: tumor nodules are labelled with black asterisks. Red asterisks denote tumor necrosis. Areas surrounded by dashed lines denote non-tumoral liver parenchyma. Scale bar: 5 mm. AFP, Alpha Fetoprotein; ARG1, Arginase 1; CD10, Cluster of Differentiation 10; CHGA, Chromogranin A; GPC3, Glypican-3; H&E, hematoxylin and eosin; HCC-NED, hepatocellular carcinoma with neuroendocrine differentiation; Hep Par-1, Hepatocyte Paraffin 1; KRT19, Keratin 19; SYP, Synaptophysin.

Supplementary Figure 3

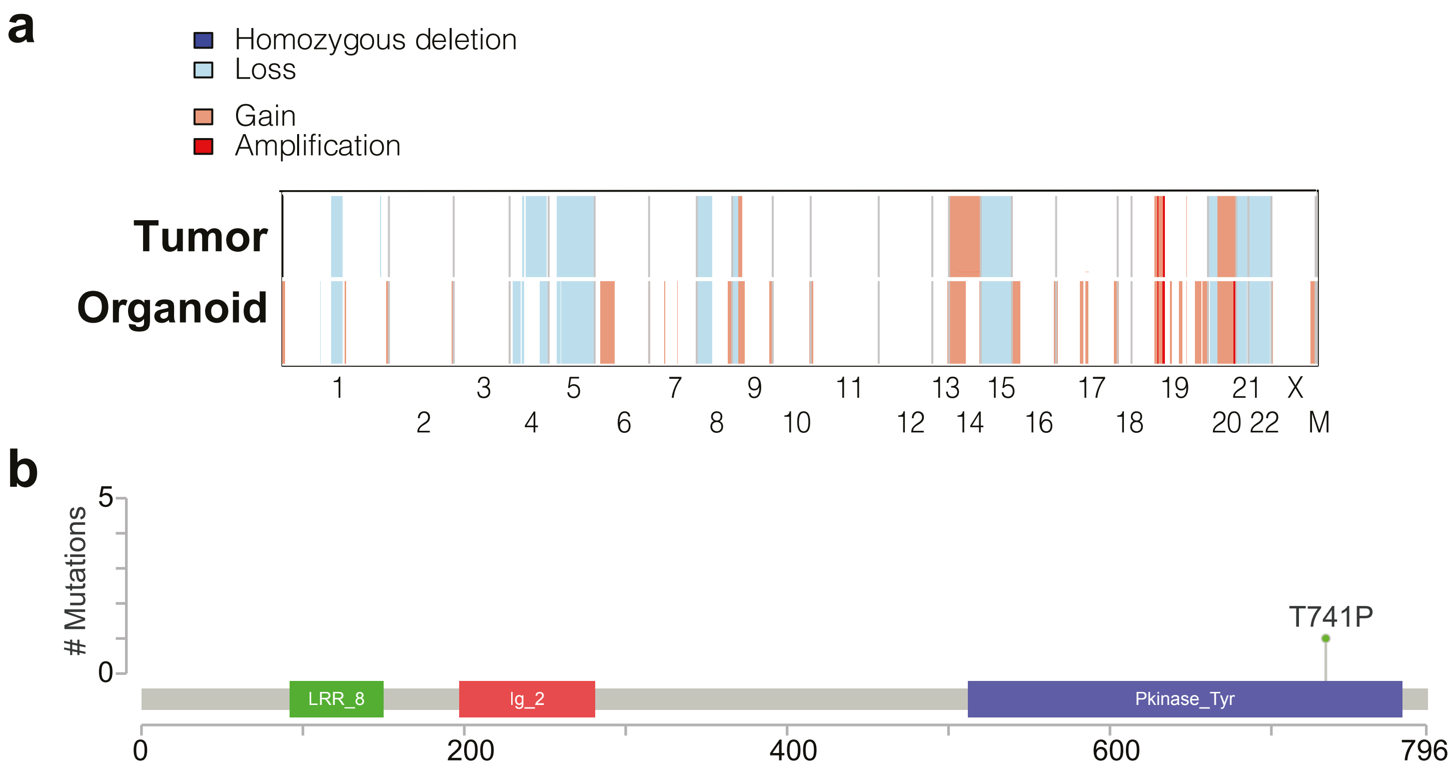

Supplementary Figure 3. Genetic characterization of HCC-NED tumor and its matched organoids. a) Summary of genome-wide copy number alterations detected by WES (whole exome sequencing). Copy number changes are colored according to the legend. **b)** Missense variant (p.T741P) in the tyrosine kinase domain of the *NTRK1* gene. HCC-NED, hepatocellular carcinoma with neuroendocrine differentiation.

Supplementary Figure 4

a

	Current treatment options for advanced HCC[1]	Current treatment options for GEP-NET[2]	Current treatment options for advanced NEC[2]	Previously described treatment options for HCC with neuroendocrine differentiation[3]
**1st line**	Azetolizumab<br>+<br>Bevacizumab	Somatostatin analogues<br>Everolimus<br>Sunitinib<br>Streptozocin + 5-FU<br>CAPTEM	Cisplatin or Carboplatin<br>+<br>Etoposide	Surgical resection<br>TACE, TARE<br>Radiation<br>Systemic CTx:<br>‣ Conventional: Cisplatin, Etoposide, Doxorubicin, 5-FU<br>‣ Kinase inhibitors: Sorafenib<br>‣ Immune checkpoint inhibitors: Nivolumab
**2nd line**	Sorafenib<br>Lenvatinib	Peptide Receptor Radionuclide Therapy	FOLFIRI<br>FOLFOX<br>CAPTEM	
**3rd line**	Regorafenib<br>Cabozantinib<br>Ramucirumab	FOLFOX<br>Temzolomide (+ Capecitabine)<br>IFNα		

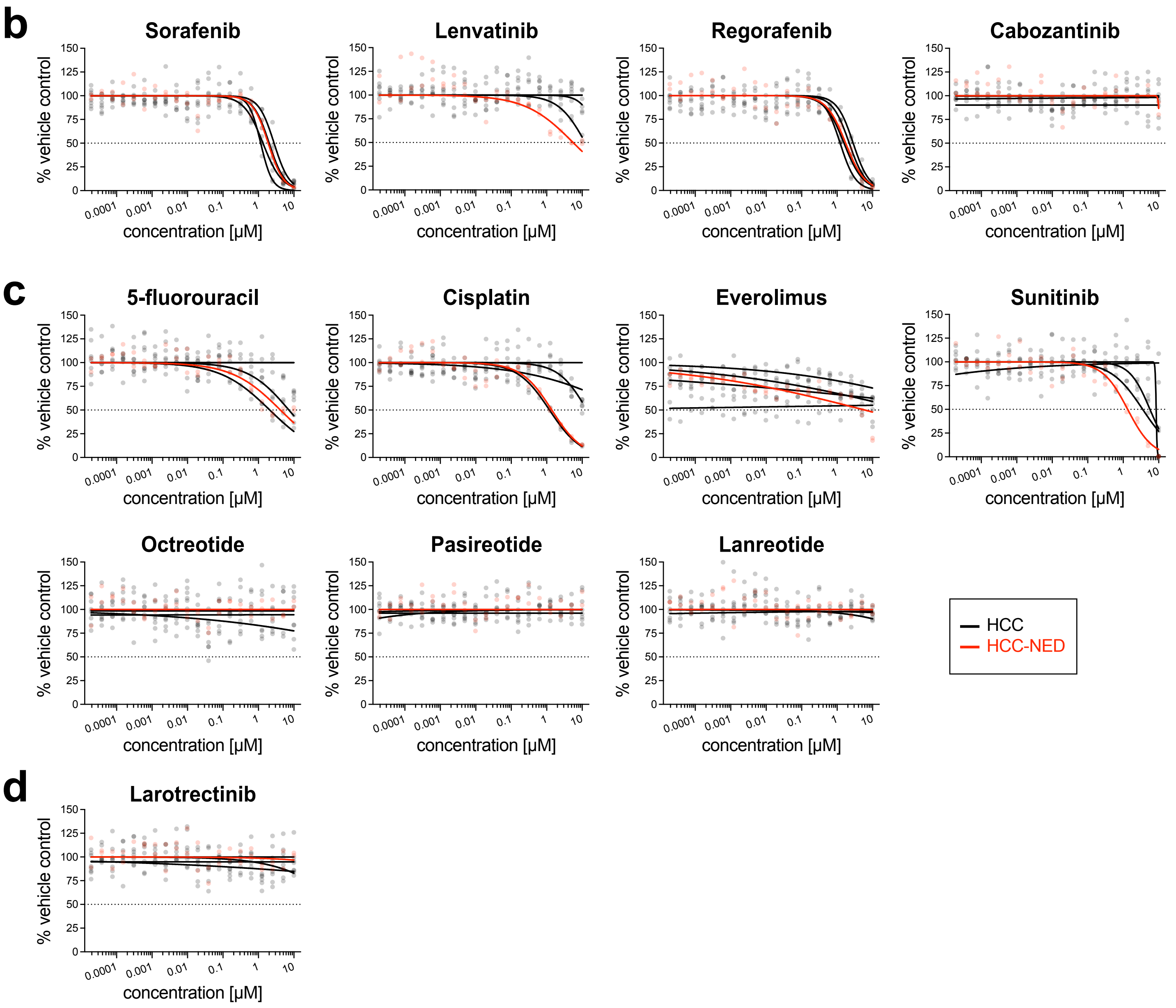

<u>Supplementary Figure 4.</u> Drug screening for HCC and NET/NEC treatment options using patient-derived organoids. a) Current treatment options approved for advanced HCC and gastroenteropancreatic (GEP-) NET/NEC as well as therapies described in case reports of patients with neuroendocrine HCCs: **[1]**according to Llovet et al.[1] and EASL Clinical Practice Guidelines[2]; **[2]**according to ESMO Clinical Practice Guidelines[3]; **[3]**according to LaRosa et al.[4], Jahan et al.[5], Nakano et al.[6], Okumura et al.[7] and Garcia et al.[8]. 5-FU, Fluorouracil; CAPTEM, Capecitabine + Temzolomide; CTx, chemotherapy; FOLFIRI, Folinic acid + 5-FU + Irinotecan; FOLFOX, Folinic acid + 5-FU + Oxaliplatin; HCC, hepatocellular carcinoma; IFNα, Interferon alpha; NEC, neuroendocrine carcinoma; NET, neuroendocrine tumor; TACE, transarterial chemoembolization; TARE, transarterial radioembolization. **b)** Dose-response curves for current treatment options in patients with advanced HCC (sorafenib, lenvatinib, regorafenib, cabozantinib). **c)** Dose-response curves for therapies recommended for gastroenteropancreatic NET/NEC (systemic chemotherapies, everolimus, sunitinib and somatostatin analogs). **d)** Dose-response curves for the pan-TRK (Tropomyosin Receptor Kinase) inhibitor larotrectinib. Concentrations ranging from 0.02 nM to 10 µM. The HCC-NED organoid line is colored in red, the four HCC organoid lines included as reference in black. After 6 days of treatment, an ATP (adenosine triphosphate)-based readout was used as a surrogate for cell number. All values were normalized to vehicle control (DMSO or water) and are displayed as the mean of n=2 biologically independent experiments. HCC-NED, hepatocellular carcinoma with neuroendocrine differentiation.

Supplementary References

1. Llovet, J.M., *et al.* Hepatocellular carcinoma. *Nat Rev Dis Primers* **7**, 6 (2021).

2. European Association for the Study of the Liver. Electronic address, e.e.e. & European Association for the Study of the, L. EASL Clinical Practice Guidelines: Management of hepatocellular carcinoma. *J Hepatol* **69**, 182-236 (2018).

3. Pavel, M., *et al.* Gastroenteropancreatic neuroendocrine neoplasms: ESMO Clinical Practice Guidelines for diagnosis, treatment and follow-up. *Ann Oncol* **31**, 844-860 (2020).

4. La Rosa, S., Sessa, F. & Uccella, S. Mixed Neuroendocrine-Nonneuroendocrine Neoplasms (MiNENs): Unifying the Concept of a Heterogeneous Group of Neoplasms. *Endocr Pathol* **27**, 284-311 (2016).

5. Jahan, N., Warraich, I., Onkendi, E. & Awasthi, S. Mixed hepatocellular carcinoma-neuroendocrine carcinoma—A diagnostic and therapeutic challenge. *Current Problems in Cancer: Case Reports* **1**(2020).

6. Nakano, A., *et al.* Combined primary hepatic neuroendocrine carcinoma and hepatocellular carcinoma: case report and literature review. *World J Surg Oncol* **19**, 78 (2021).

7. Okumura, Y., *et al.* Combined primary hepatic neuroendocrine carcinoma and hepatocellular carcinoma with aggressive biological behavior (adverse clinical course): A case report. *Pathol Res Pract* **213**, 1322-1326 (2017).

8. Garcia, M.T., Bejarano, P.A., Yssa, M., Buitrago, E. & Livingstone, A. Tumor of the liver (hepatocellular and high grade neuroendocrine carcinoma): a case report and review of the literature. *Virchows Arch* **449**, 376-381 (2006).
