# Supplementary material for: Patient-derived tumor organoids for personalized medicine in a patient with rare hepatocellular carcinoma with neuroendocrine differentiation: a case report
Source: Commun Med (Lond). 2022 Jul 1;2:80. doi: 10.1038/s43856-022-00150-3 (PMC9249908; doi:10.1038/s43856-022-00150-3)
Supplement: Supplementary file 8 — Reporting Summary [file 43856_2022_150_MOESM8_ESM.pdf]

nature research

Corresponding author(s): Markus Heim, Salvatore Piscuoglio

Last updated by author(s): Oct 6, 2021

Reporting Summary

Nature Research wishes to improve the reproducibility of the work that we publish. This form provides structure for consistency and transparency in reporting. For further information on Nature Research policies, see our Editorial Policies and the Editorial Policy Checklist.

Statistics

For all statistical analyses, confirm that the following items are present in the figure legend, table legend, main text, or Methods section.

n/a	Confirmed	
☐	☒	The exact sample size (*n*) for each experimental group/condition, given as a discrete number and unit of measurement
☐	☒	A statement on whether measurements were taken from distinct samples or whether the same sample was measured repeatedly
☐	☒	The statistical test(s) used AND whether they are one- or two-sided<br>*Only common tests should be described solely by name; describe more complex techniques in the Methods section.*
☒	☐	A description of all covariates tested
☒	☐	A description of any assumptions or corrections, such as tests of normality and adjustment for multiple comparisons
☒	☐	A full description of the statistical parameters including central tendency (e.g. means) or other basic estimates (e.g. regression coefficient) AND variation (e.g. standard deviation) or associated estimates of uncertainty (e.g. confidence intervals)
☒	☐	For null hypothesis testing, the test statistic (e.g. *F*, *t*, *r*) with confidence intervals, effect sizes, degrees of freedom and *P* value noted<br>*Give P values as exact values whenever suitable.*
☒	☐	For Bayesian analysis, information on the choice of priors and Markov chain Monte Carlo settings
☒	☐	For hierarchical and complex designs, identification of the appropriate level for tests and full reporting of outcomes
☒	☐	Estimates of effect sizes (e.g. Cohen's *d*, Pearson's *r*), indicating how they were calculated

Our web collection on statistics for biologists contains articles on many of the points above.

Software and code

Policy information about availability of computer code

Data collection	Whole exome sequencing: Illumina NovaSeq 6000
Data analysis	Whole exome sequencing: BWA version 0.7.12, GATK version 4.1, Picard, GATK 4.1.4.1, strelka v.2.9.10, FACETS v.0.5.14<br>Drug screening: Prism8 (GraphPad Software Inc)

For manuscripts utilizing custom algorithms or software that are central to the research but not yet described in published literature, software must be made available to editors and reviewers. We strongly encourage code deposition in a community repository (e.g. GitHub). See the Nature Research guidelines for submitting code & software for further information.

Data

Policy information about availability of data

All manuscripts must include a data availability statement. This statement should provide the following information, where applicable:

- Accession codes, unique identifiers, or web links for publicly available datasets
- A list of figures that have associated raw data
- A description of any restrictions on data availability

All data that support the study and summarized in the manuscript will be available from the corresponding author upon request. WES data will be uploaded to a public repository.

Field-specific reporting

Please select the one below that is the best fit for your research. If you are not sure, read the appropriate sections before making your selection.

☒ Life sciences ☐ Behavioural & social sciences ☐ Ecological, evolutionary & environmental sciences

For a reference copy of the document with all sections, see nature.com/documents/nr-reporting-summary-flat.pdf

Life sciences study design

All studies must disclose on these points even when the disclosure is negative.

Sample size	No statistical methods were used to predetermine the sample size. The sample size was based on previous experience and feasibility of experiments.
Data exclusions	No data were excluded.
Replication	Experiments were independently repeated as indicated in the supplementary materials and methods and could have been successfully replicated.
Randomization	HCC organoids serving as reference in our drug screening were selected randomly from our HCC organoid biobank.
Blinding	Experiments did not use blinding, as our analysis does not rely on subjective measures.

Reporting for specific materials, systems and methods

We require information from authors about some types of materials, experimental systems and methods used in many studies. Here, indicate whether each material, system or method listed is relevant to your study. If you are not sure if a list item applies to your research, read the appropriate section before selecting a response.

Materials & experimental systems

n/a	Involved in the study	
☐	☒	Antibodies
☒	☐	Eukaryotic cell lines
☒	☐	Palaeontology and archaeology
☐	☒	Animals and other organisms
☐	☒	Human research participants
☒	☐	Clinical data
☒	☐	Dual use research of concern

Methods

n/a	Involved in the study	
☒	☐	ChIP-seq
☒	☐	Flow cytometry
☒	☐	MRI-based neuroimaging

Antibodies

Antibodies used	AFP (Ventana Cat. No. 760-2603), ARG1 (Ventana Cat. No. 760-4801), CD10 (Ventana Cat. No. 790-4506), CD56 (Ventana Cat. No. 790-4465), CHGA (Ventana Cat. No. 670-2509), GPC3 (Ventana Cat. No. 790-4564), HLA-ABC (Abcam Cat. No. ab70328), Hep Par-1 (Ventana Cat. No. 760-4350), KRT19 (Ventana Cat. No. 760-4281), Ki-67 (Dako Cat. No. IR626), SYP (Ventana Cat. No. 790-4407), and SSTR2 (Abcam Cat. No. ab134152)
Validation	Antibody were performed by suppliers and are available on the manufacturer's website.

Animals and other organisms

Policy information about studies involving animals; ARRIVE guidelines recommended for reporting animal research

Laboratory animals	Immunodeficient Non-obese diabetic (NOD) Severe combined immunodeficiency (SCID) Gamma (NSG) mice (The Jackson Laboratory)
Wild animals	The study did not involve wild animals.
Field-collected samples	The study did not involve samples collected from the field.
Ethics oversight	Animal Care Committee of the Canton Basel-Stadt, Switzerland

Note that full information on the approval of the study protocol must also be provided in the manuscript.

Human research participants

Policy information about studies involving human research participants

Population characteristics	Patient characteristics are described in the text and Table S2.
Recruitment	Patients undergoing diagnostic liver biopsy or liver surgery at the University Hospital of Basel
Ethics oversight	The study was carried out in accordance with The Code of Ethics of the World Medical Association (Declaration of Helsinki) and was approved by the Ethics Committee of North Western Switzerland (Authorization numbers EKNZ 2014-099 and BASEC 2019-02118). Written informed consent was obtained from all patients enrolled in this study.

Note that full information on the approval of the study protocol must also be provided in the manuscript.
